# Longitudinal Evaluation of PostPandemic Electroconvulsive Therapy Services at Birmingham and Solihull Mental Health NHS Foundation Trust

**DOI:** 10.1192/bjo.2026.11593

**Published:** 2026-06-30

**Authors:** Vishi Sachdeva, Gabriel Kogo, Kishan Pankhania, Taleaa Mohsin, Dhruba Bagchi

**Affiliations:** 1 East London NHS Foundation Trust, London, United Kingdom; 2 Birmingham and Solihull NHS Mental Health Foundation Trust, Birmingham, United Kingdom

## Abstract

**Aims:**

- To evaluate the referral patterns and uptake of the ECT service in relation to patient demographics and diagnosis.
- To assess referrers’ experiences of the ECT referral process and identify barriers influencing referral quality and access.
- To determine patient outcomes following ECT and evaluate the overall effectiveness of this treatment modality.

**Methods:**

A retrospective longitudinal design was used. Case records for all patients referred for ECT between 01/04/2022 and 31/03/2023 were reviewed, forming a complete 12 month cohort. Demographic, clinical, and referral information was extracted from electronic patient records, and each case was followed for two years to assess longerterm outcomes. Data were collated using a structured Microsoft Excel tool prior to analysis.

**Results:**

Seventy patients were referred during the evaluation period. Forty nine completed treatment (69%), eight declined despite being assessed as suitable, and twelve referrals were screened out–primarily due to administrative errors where ECG referrals were mistakenly submitted as ECT requests. Referrals were predominantly female (48 vs. 22 males), with most patients aged 55 or older. Ethnicity analysis showed a majority of White British patients (n=48), with lower representation from minority ethnic groups. Most referrals originated from the MHSOP inpatient acute team (n=26). Waiting time analysis showed that 36.2% of patients waited more than 10 days from referral to treatment. Diagnostic profiles were dominated by severe depressive episodes, particularly psychotic depression (n=19). Short term outcomes were strong, with 73.5% maintaining improvement at one month. The highestrelapse risk occurred between 1–6 months, with maintained improvement falling to 49% at six months. At two years, 51% remained well, although attrition increased to 20.4%.

**Conclusion:**

A robust continuation plan is needed between the 1 to 6 months post ECT which is consistent with known patterns where ECT response can fade. Also in practical terms, the post ECT outcome data points to it being effective for acute episodes, but maintenance of gains is not automatic and depends on timely continuation pharmacotherapy, structured relapse-prevention planning, psychological and social supports, and for some patients, continuation/maintenance ECT. Service level findings indicate opportunities to improve referral accuracy, reduce pathway delays, address demographic disparities, and strengthen patient education and referrer training to enhance accessibility, efficiency, and clinical effectiveness

